# COVID-19 and the role of 3D printing in medicine

**DOI:** 10.1186/s41205-020-00064-7

**Published:** 2020-04-27

**Authors:** Rance Tino, Ryan Moore, Sam Antoline, Prashanth Ravi, Nicole Wake, Ciprian N. Ionita, Jonathan M. Morris, Summer J. Decker, Adnan Sheikh, Frank J. Rybicki, Leonid L. Chepelev

**Affiliations:** 1 Centre for Additive Manufacture, Royal Melbourne Institute of Technology, School of Engineering, 58 Cardigan St, Carlton, Melbourne, VIC 3001 Australia; 2 Department of Physical Sciences, Victorian Comprehensive Cancer Centre, Peter MacCallum Cancer Centre, Level B1/305 Grattan St, Melbourne, VIC 3000 Australia; 3 Division of Cardiology, Cincinnati Children’s Hospital Medical Center, 3333 Burnet Avenue MLC 2003, Cincinnati, OH 45229-3039 USA; 4 Department of Mechanical and Materials Engineering, College of Engineering and Applied Science, University of Cincinnati, Cincinnati, OH 45219 USA; 5 Department of Radiology, University of Cincinnati College of Medicine, 234 Goodman Street, Cincinnati, OH 45267 USA; 6 Department of Radiology, Montefiore Medical Center, Albert Einstein College of Medicine, 111 East 210th Street, Bronx, NY 10467 USA; 7 Department of Biomedical Engineering, Jacobs School of Medicine and Biomedical Sciences at the University at Buffalo, University at Buffalo School of Engineering and Applied Sciences, 8052 Clinical Translational Research Center, 875 Ellicott Street, Buffalo, NY 14203 USA; 8 Anatomic Modeling Lab, Department of Radiology, Division of Neuroradiology, Mayo Clinic, 200 First St. SW, Rochester, MN 55905 USA; 9 Department of Radiology, USF Health Morsani College of Medicine, Tampa, FL 33606 USA; 10 Department of Radiology, University of Ottawa School of Medicine, 501 Smyth Road, Ottawa, ON K1H 8L6 Canada; 11 Department of Radiology, University of Cincinnati, University of Cincinnati Medical Center, 234 Goodman Street, Cincinnati, OH 45219 USA

As of March 12, 2020, the World Health Organization classified coronavirus disease 2019 (COVID-19) as a pandemic, at the time of writing affecting nearly every country and territory across the globe [1]. During this time of social and economic despair, global healthcare systems are under critical strain due to severe shortages of hospital beds and medical equipment. Patients with COVID-19, the disease caused by severe acute respiratory syndrome coronavirus 2 (Fig. 1), are at risk for acute respiratory distress syndrome (ARDS) and a fraction will require high-level respiratory support to survive [2]. Additionally, significant strain has been placed on personal protective equipment (PPE) supplies required to protect the healthcare workers helping to treat critically ill patients during this pandemic. At the time of writing, there are active disruptions of medical supply chains throughout Europe and in the United States at the hospital level, particularly in the states of New York and Washington.

**Fig. 1:**
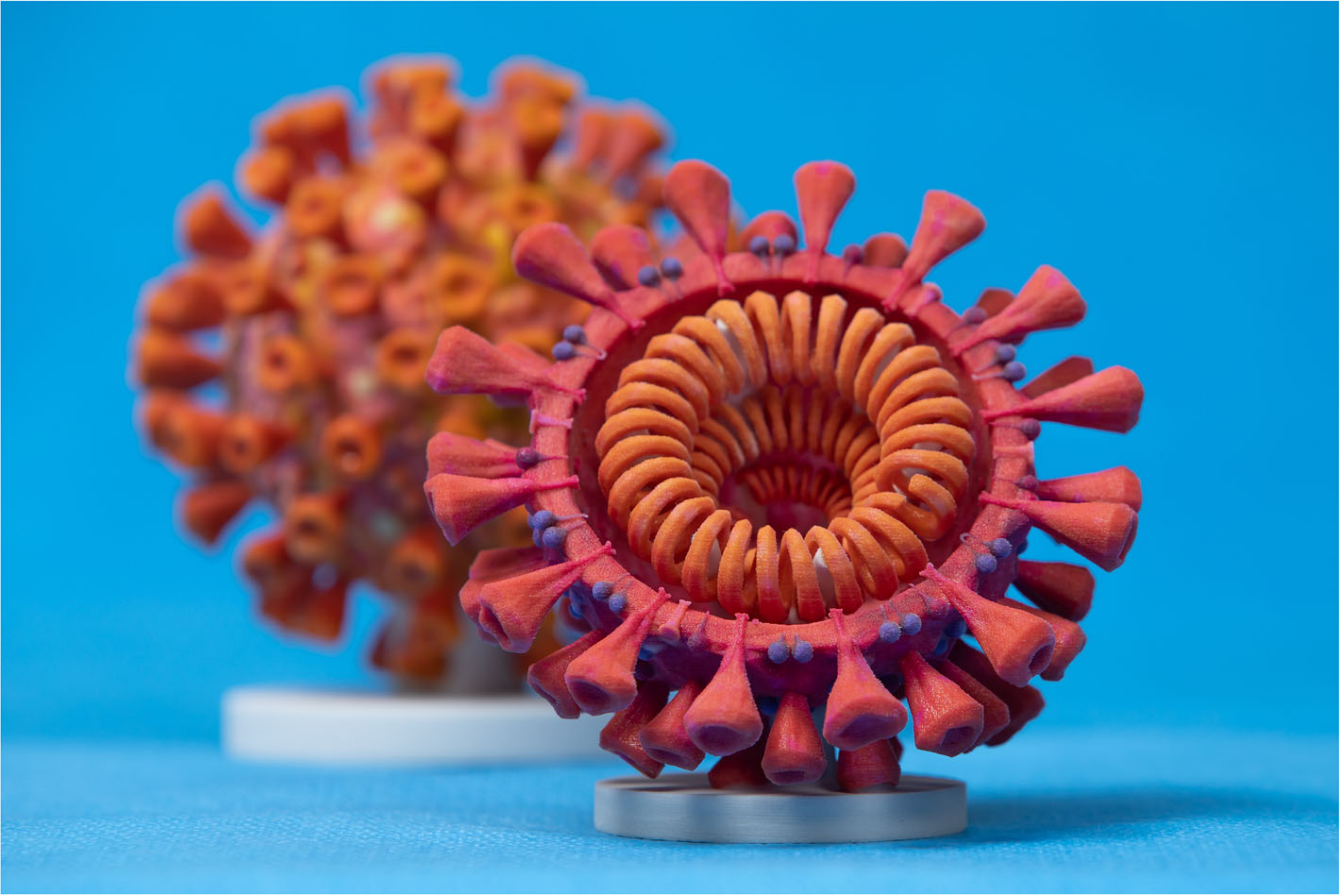
3D printed model of severe acute respiratory syndrome coronavirus 2

The purpose of this Editorial is to highlight recent (as of April 1, 2020) initiatives and collaborations performed by companies, hospitals, and researchers in utilising 3D printing during the COVID-19 pandemic and to support local 3D printing efforts that can be lifesaving. The 3D printing community can refocus its medical attention internationally, capitalizing on centralized large-scale manufacturing facilities as well as locally distributed manufacturing of verified and tested CAD files. In addition, there are multiple medical, engineering, and other societies and groups that can pull together to work on common needs, many of which are outlined in this Editorial.

While models discussed here are primarily open-source necessities available at the time of writing, the CAD file resources referred to in this Editorial are intended for a discussion of an evolving collection of ready-to-print models and links to the relevant resources to aid in supporting urgent medical response. An example collection can be found at the NIH 3D Print Exchange.

We must acknowledge that at the time of writing, the clinical effectiveness of many of the devices manufactured according to the CAD files described in this Editorial has not been tested and many of these devices have not been approved for frontline clinical use by relevant regulatory bodies. The authors of this Editorial cannot guarantee clinical effectiveness of the presented devices and would urge consideration of these resources at the users’ discretion and only where no medically cleared alternatives are available.

## Hospital respiratory support apparatus

The recent impact of COVID-19 in Italy has caused regional shortages of key equipment, including masks and hoods for non-invasive ventilation in CPAP/PEEP respiratory support. Crucially, venturi valves, key components of such respiratory support equipment [3] proved difficult to reproduce or substitute in the setting of these shortages. While venturi valve design is subject to copyright and patent covers, certain emergencies resulting in life-or-death decisions may justify full use regardless of intellectual property, in the appropriate clinical setting. This critical demand has resulted in the 3D printing community of physicians and engineers at a local Italian startup Isinnova successfully developing methods for manufacturing these valves to bolster local supply [4]. Additional methods of bolstering local ventilator supply include the use of a single ventilator for multiple patients with a 3D printed ventilator splitter. Fortunately, the US FDA does not object to the creation and use of certain devices such as the T-connector that meet specifications described in the instructions provided to the FDA for use in placing more than one patient on mechanical ventilation when the number of patients who need invasive mechanical ventilation exceeds the supply of available ventilators and the usual medical standards of care have been changed to crisis care in the interest of preserving life. The FDA's "no objection" policy in this regard applies during the duration of the declared COVID-19 emergency.

**Fig. 2:**
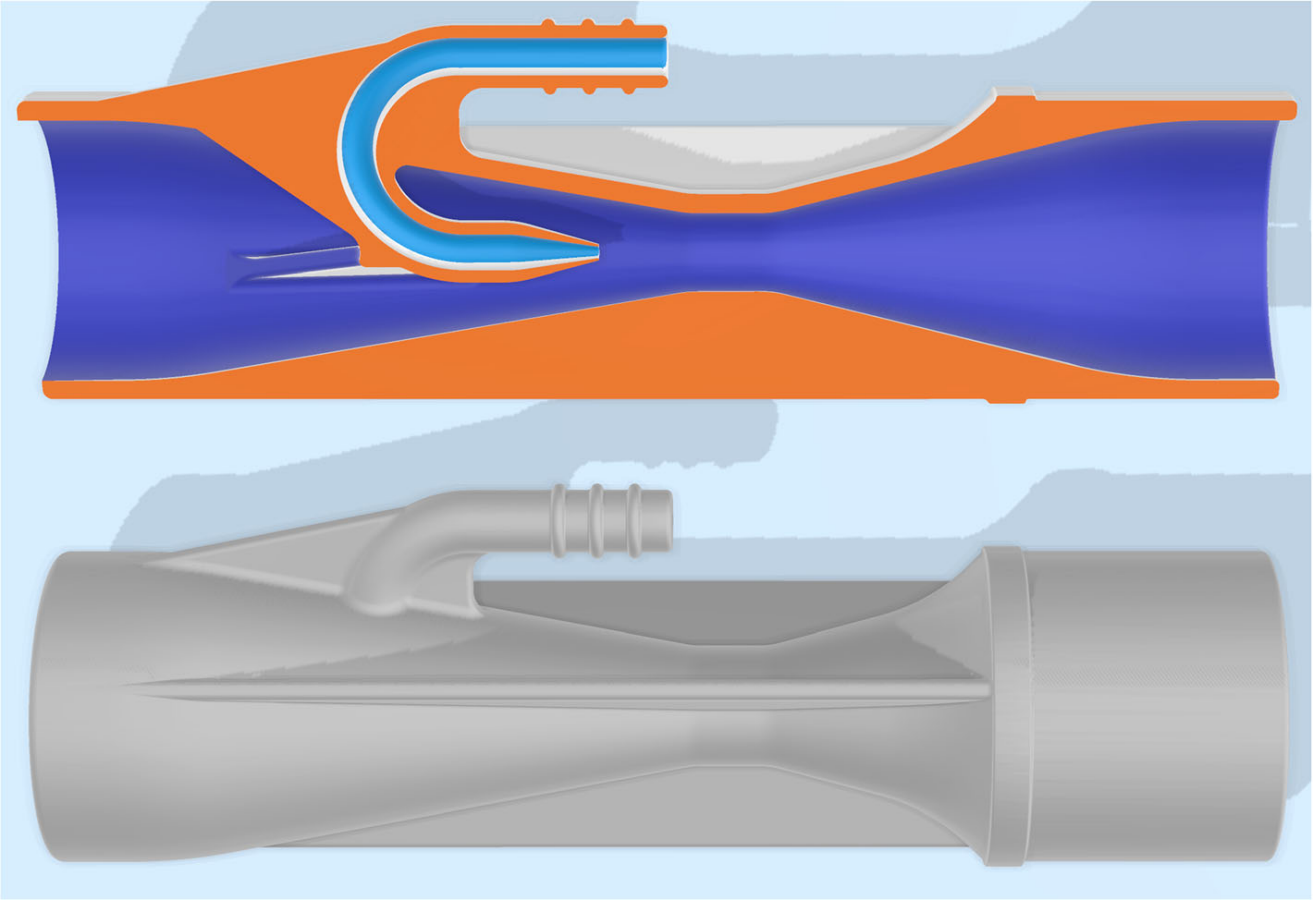
Open-source non-adjustable Venturi valve design for an FiO2 of 33% at supplemental oxygen flow rate of 10 l per minute. The cross-sectional view (above) demonstrates the inner structure of this device with a small oxygen port (light blue) and a larger air intake (left)

Access to such models is still limited for many local 3D printing community members and will require close collaboration between companies and hospitals to ensure adequate manufacturing approaches and appropriate clinical use. The reverse-engineered 3D printable model of the Isinnova valve is not widely available at the time of writing, with the authors maintaining the position that such resources should be adequately evaluated and used only when such equipment is not available from the original manufacturers. Ongoing efforts by the engineers at Isinnova are focusing on developing creative adaptations of existing products for respiratory support, for example by adapting a snorkelling mask into a non-invasive ventilator [5]. Most recently, non-adjustable venturi valve designs were developed and made available by the GrabCAD user Filip Kober [6]. These valve designs achieve specific levels of inspired oxygen (FiO_2_) at set rates of supplemental oxygen supply (Fig. 2). Model porosity may inadvertently alter intended FiO_2_ levels, requiring the use of printing technologies that ensure airtight parts.

Automated ventilators with flow-driven, pressure-controlled respiratory support systems featuring safety valves, spontaneous respiration valves, and flexible membranes present an ongoing open source design challenge with some promising results, including the Illinois RapidVent design. While sourcing ventilators and ventilator parts from existing manufacturers is the clearly preferred option when feasible, the supply of these crucial devices is inadequate in many areas. A solution currently being applied to this challenge in Europe and the United States is the creation of 3D printed ventilator splitters and adjustable flow control valves, such as the no2covid-ONE valve, to be able to adapt a single ventilator for use with multiple patients who have different oxygen requirements [7]. We anticipate new creative solutions for such increasingly complex challenges from emerging international open source design efforts such as the Montreal General Hospital Foundation Code Life Ventilator Challenge [8] as the COVID-19 health crisis emerges.

## Personal protective equipment (PPE)

Quarantine measures in the setting of this pandemic have sparked tension and fear among the lay public. An unfortunate consequence of this is unnecessary panic buying, leaving those who need these products, such as health care workers, in limited supply. Members of the global 3D printing community have designed a plethora of reusable personal protective equipment devices with insertable filters, primarily manufactured using low-cost desktop filament extrusion printers. To our best knowledge, PPE items in need at the time of writing include splash-proof face shields, surgical masks, N95 masks, N90 masks, powered air-purifying respirator (PAPR) hoods, and Controlled Air Purifying Respirator hoods (CAPR).

Many of the PPE designs highlighted here are works in progress, and the effectiveness of locally manufactured derivatives of these devices should be carefully evaluated locally. Additionally, these PPEs are intended to be reusable, and therefore local manufacturing efforts should carefully consider compatibility with the available sterilization techniques and the condition of all PPE devices should be monitored following sterilization on an ongoing basis.

To ensure the best fit, personalizing these masks may be achieved by printing in several sizes, experimenting with flexible materials, or surface scanning intended users’ faces and carrying out additional CAD to virtually fit these masks on an individual basis [9]. While this individualized approach may limit manufacturing throughput, the improved functionality may justify this impact on throughput.

In general, throughput may be the most challenging factor to address in developing 3D printed PPE in smaller-scale local 3D printing laboratories. Many of the models highlighted here require several hours to print on conventional desktop printers. While many 3D printing laboratories can parallelize this process with multiple printers, throughput will likely remain limited to dozens of masks per printer and 3D printing resources should therefore be assigned judiciously.

### Face masks

This section refers to PPE used to protect the wearer from airborne particles and liquid contaminants on the face. For the purpose of this article these are referred to as "face masks" and there are several 3D printed solutions. The FDA, NIH 3D Print Exchange, and the United States Veterans' Association are working together in this regard, including developing a prototype N95 mask currently being tested. In the meantime, numerous face mask designs have been proposed and tested by individual users, researchers, physicians, and commercial entities alike with variable degrees of success. In all cases, the end users must clearly understand that only prototypes are available at this point and local testing procedures, potentially modified from established routine N95 fit testing, are crucial to assess the quality of PPE.

**Fig. 3:**
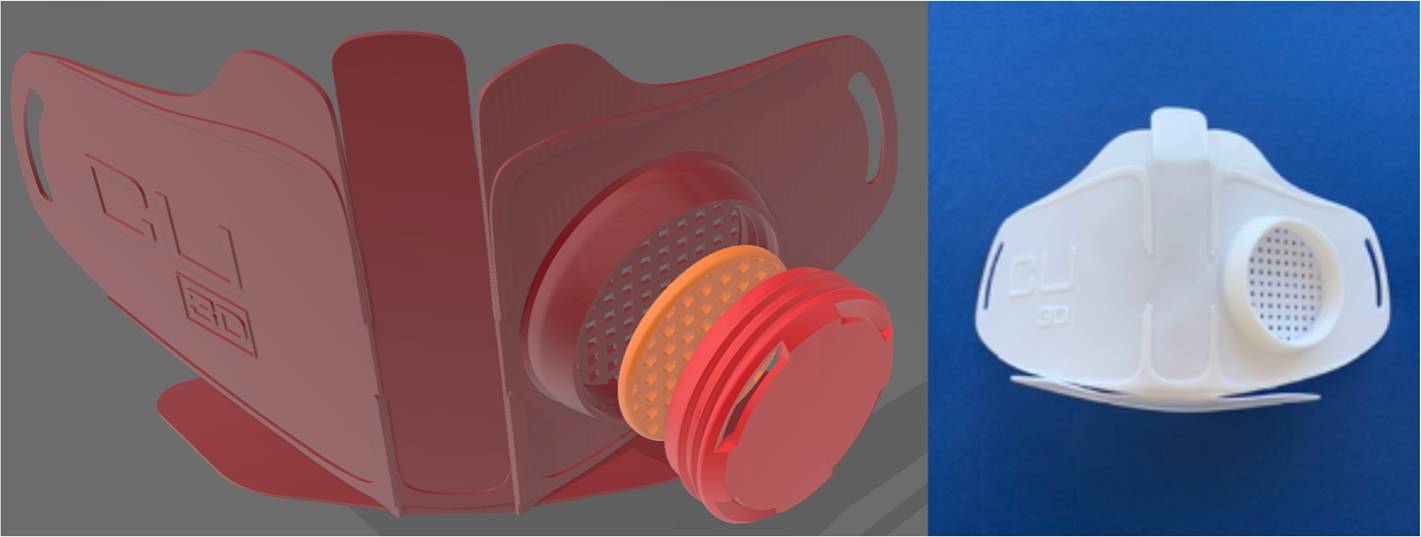
Copper3D NanoHack mask model demonstrating an intermediate stage in mask completion, left. A 3D printed model of this mask, right

The Copper3D NanoHack mask [10] demonstrates the limitations of the community-generated designs and the need for design improvements based on local testing and available technical base. This mask can be printed with Polyactic Acid (PLA) filament as a flat piece, and is intended to be subsequently manually assembled into its final three-dimensional configuration after heating to a temperature of 55–60 °C (131–140 °F) via forced hot air (e.g. a hairdryer) or by submerging it in hot water (Fig. 3). Crucially, all seams must be manually sealed to ensure an airtight fit. The mask includes a simple air intake port into which two reusable filters may be inserted, with a screw-in cover to hold the filters in place. This design has several drawbacks. Due to the flat design, only one mask can be printed at a time on most desktop printers, limiting throughput. Practically, our initial tests demonstrate difficulties folding these masks created using conventional PLA filament, with significant gaps along the seams that are difficult to mitigate. If successfully sealed, the mask may provide limited airflow for some users and a second breathing port, achievable by mirror-imaging the port-bearing half of the mask, may need to be added. As a result of multiple limitations, this mask is currently undergoing revisions by the original designer.

**Fig. 4:**
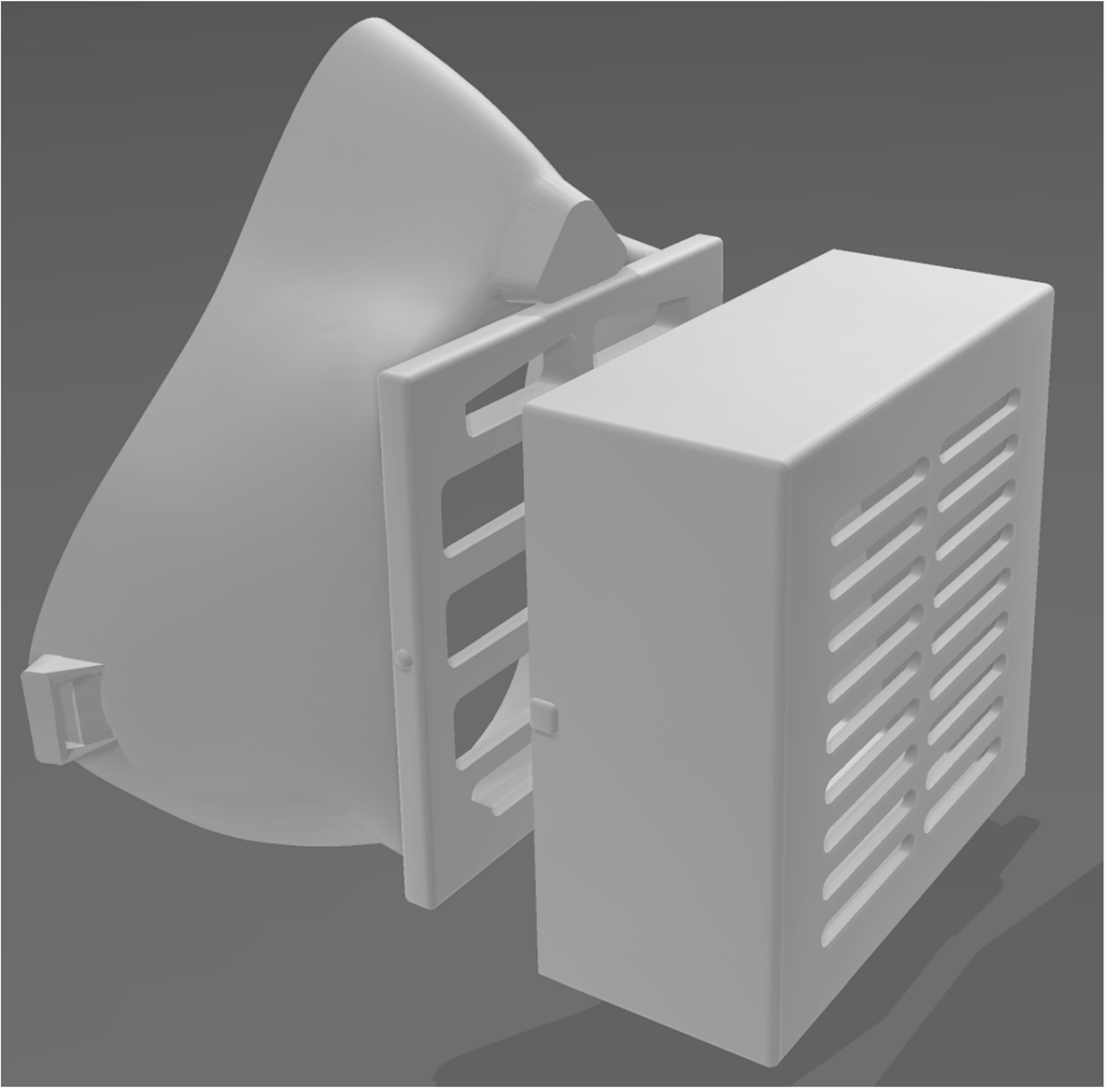
HEPA mask design with a box for HEPA filter insertion

**Fig. 5:**
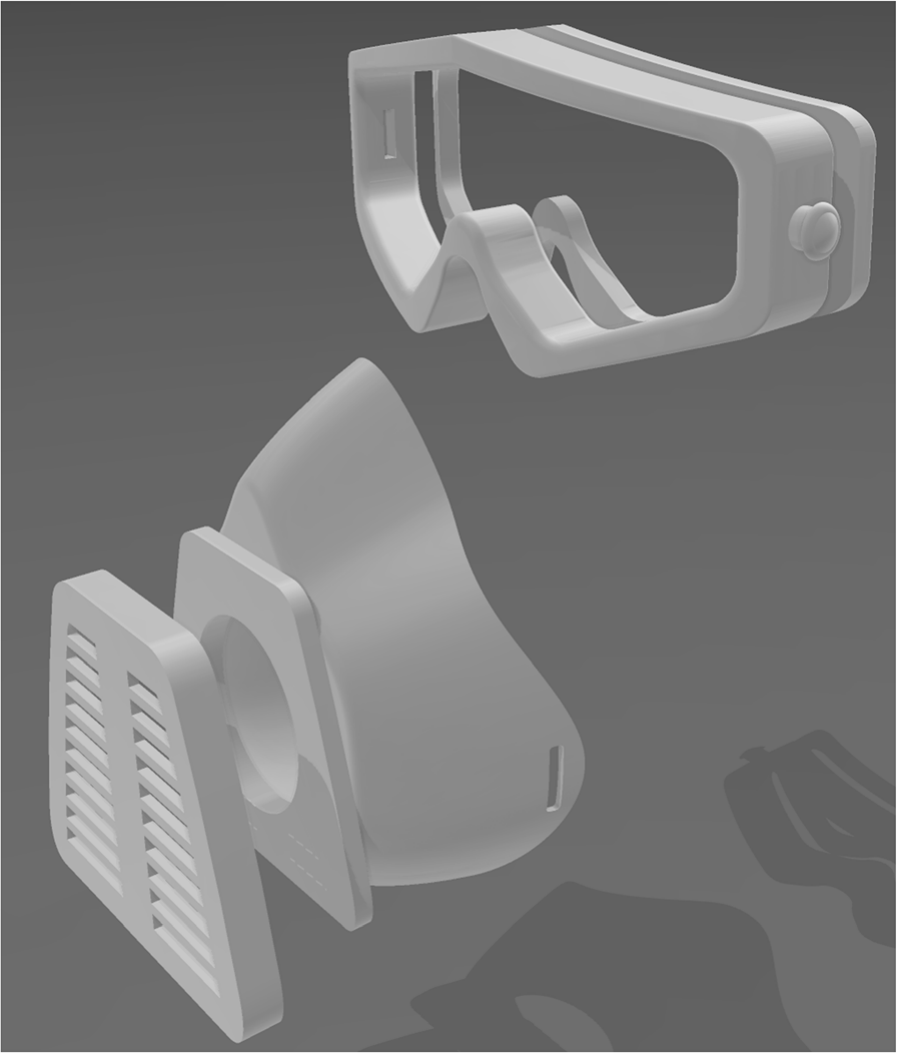
Creality mask and goggle design

The HEPA mask designed by the Thingiverse user Kvatthro [11] may be manufactured using most desktop printers. PLA filament is suggested due to the possibility of fitting the mask to the individual user after heat exposure, which is important to ensure the best possible air seal in field conditions. The mask comes in male and female variants and allows space for an exchangeable HEPA filter insert within a port at the front of the mask (Fig. 4). A similar design has been proposed by the Chinese company Creality [12], with a different configuration of the filter holder, intended for insertion of layers of folded fabric or filters (Fig. 5). The Creality goggles require separately sourcing transparent plastic inserts, which may be obtained from repurposed household items. As with all masks, judicious testing for seal adequacy and experimentation with sizing and materials are required for implementation.

**Fig. 6:**
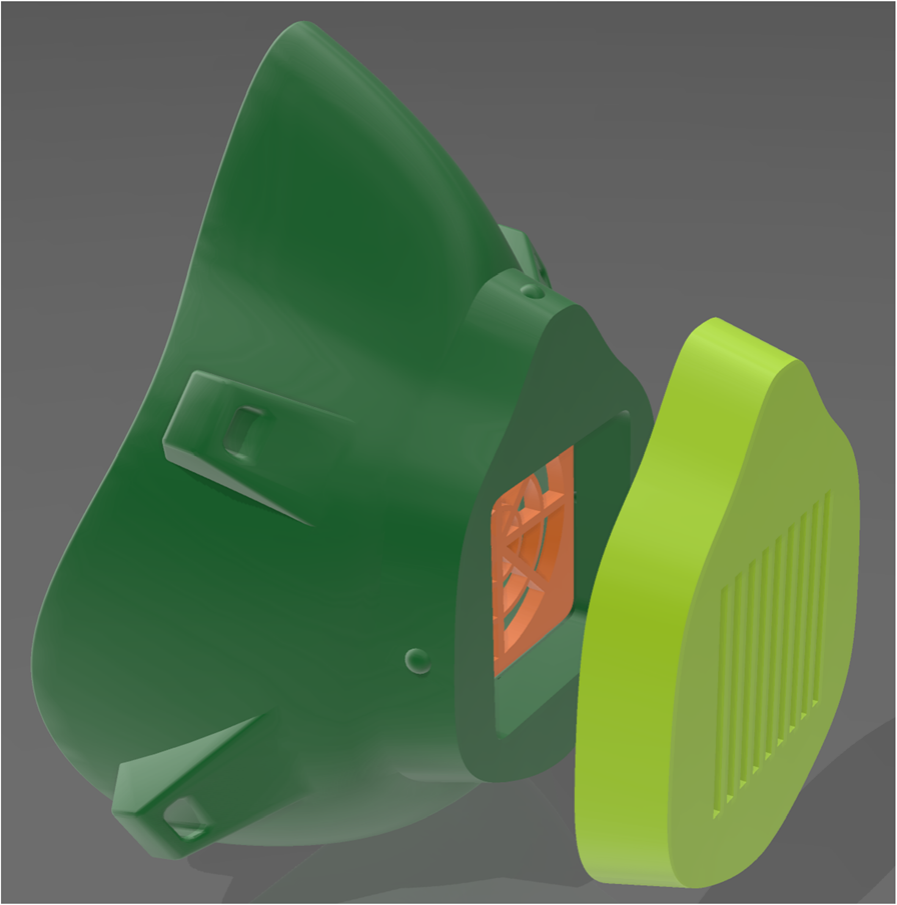
Lowell Makes mask design, with the filter held in place by a grid on the inside of the mask and a cap on the front of the mask

The Lowell Makes mask is a variant of the replaceable front filter design which offers the benefit of printing without supports or adhesion [13] (Fig. 6). The mask is intended to be lined with a foam padding on the inside. While addition of elements such as foam padding to reusable PPE like the Lowell Makes mask improves user comfort, this may impact the selection of sterilization approaches and must be considered carefully.

Finally, additional creative designs, such as the “Flexible Mask Valvy” by the Thingiverse user iczfirz [14] have demonstrated the feasibility of printing PLA masks on a cloth bed platform. This design allows for reusability with dedicated filter inserts.

### Face shields

**Fig. 7:**
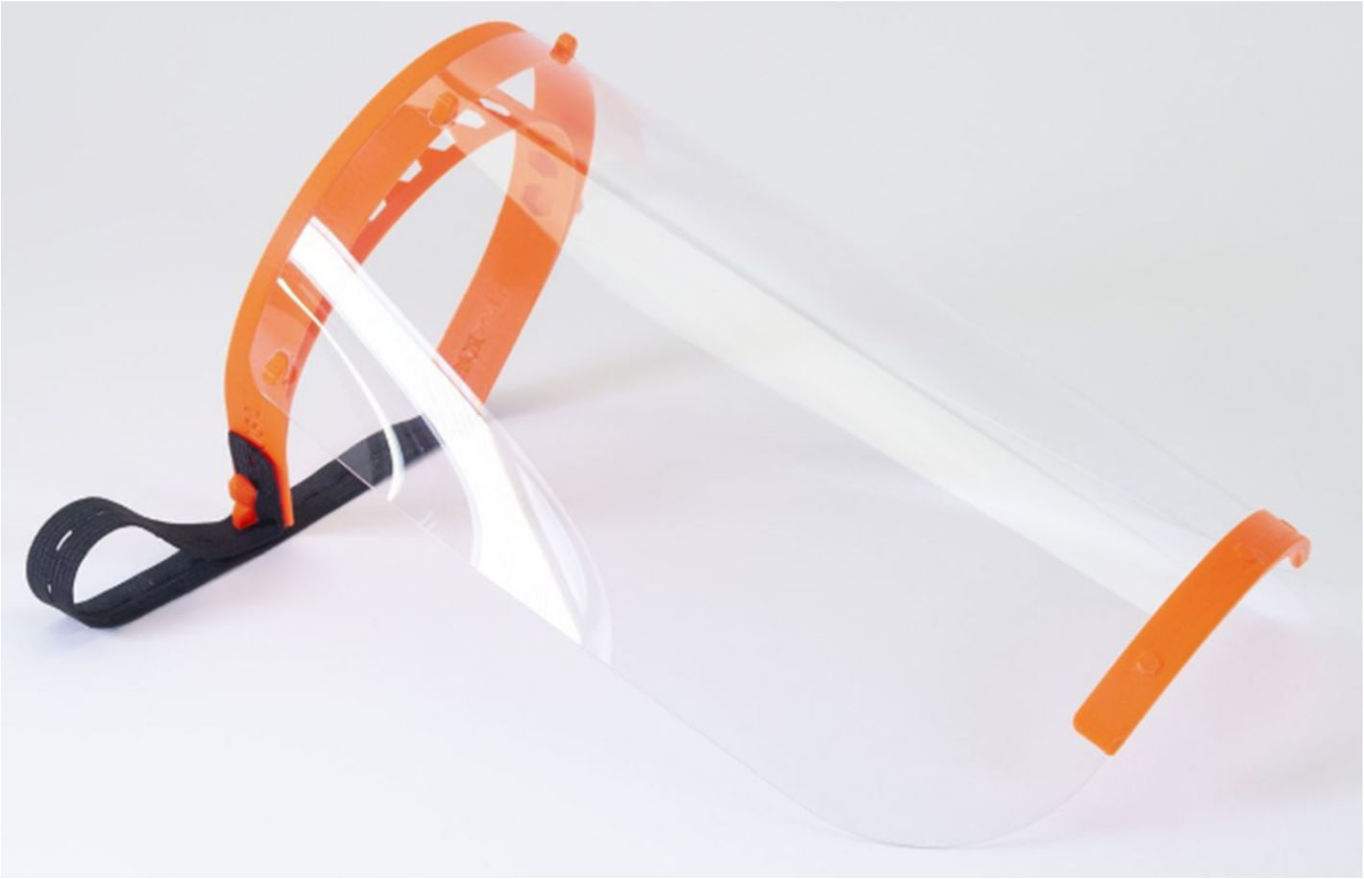
Early reusable Prusa Research 3D printed headband allowing the insertion of flexible shields. Image modified from [15]

Additional variations on personal protective equipment include protective face shields, such as those designed by Prusa [15]. These simple devices feature a reusable printable headpiece to which a separately sourced transparent sheet of plastic can be attached to create a face shield, protecting the user’s eyes and mouth (Fig. 7). Face shield designs completely bypassing 3D printing have also emerged.

## Environmental solutions

**Fig. 8:**
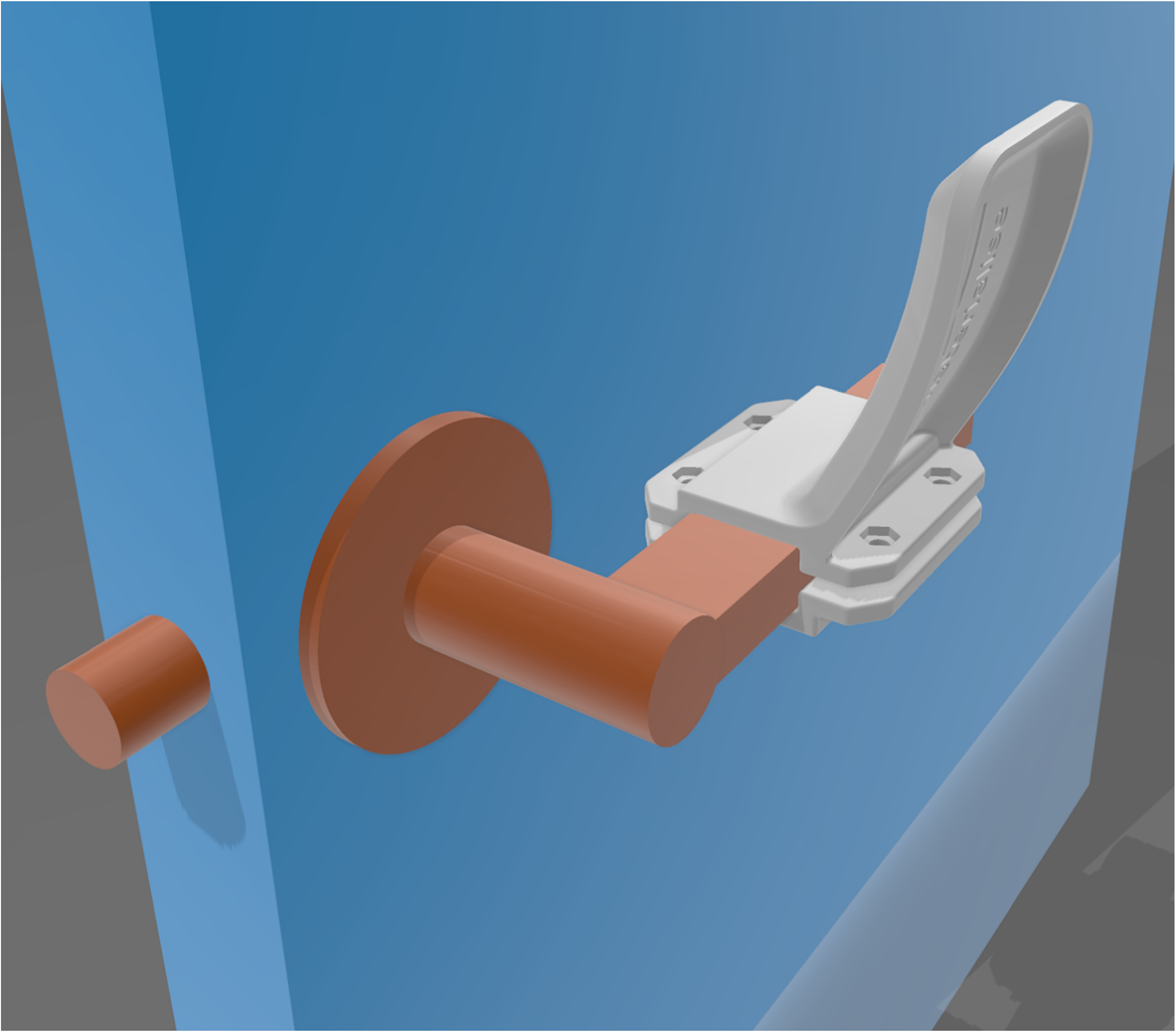
Ready to print door handle model by Materialise. The handle is operated by placing one’s sleeved hand on the surface of the handle closest to the door and applying pressure down and towards the operator

COVID-19 requires meticulous precautions in limiting person-to-person spread via direct contact with objects or surfaces such as door handles. Simple interventions limiting such transmission can have far-reaching consequences. Transmission from door handles may be problematic in public and in medical centers which usually have a large number of doors designed for patient privacy or ward control, especially during periods of isolation during pandemics. While meticulous and regular surface cleaning partially addresses this issue, modifications of a range of handles to allow alternative mechanisms for opening doors without direct skin-to-surface contact have been recently developed at Materialise. These ready to print door handle accessories [16] can be manufactured on most 3D printing platforms (Fig. 8).

## Printed equipment disinfection

Current CDC guidelines for disinfection and sterilization in healthcare facilities define three major levels of pathogen eradication: cleaning, disinfection, and sterilization [17]. Cleaning is defined as removing visible soil and organic material. The definition of disinfection varies based on whether it is low or high level, and refers to removing many or all microorganisms respectively, under optimal conditions. Sterilization is defined as assured complete eradication of all microbial life on a given piece of equipment.

Based on analogy with the established operational parameters for reusable respirators [18], high-level disinfection is likely the most appropriate modality when dealing with reusable 3D printed personal protective equipment. Recommended disinfection agents range from concentrated alcohol to quaternary ammonium compounds, and the precise agent selection would likely vary depending on the utilized material and printing technology. Initial testing of the preferred/available disinfection mode may be conducted prior to scaling up manufacture, in consultation with local hospital policies and consideration of disinfection material availability. Ensuring compatibility with widely available common household chlorine-based or hydrogen peroxide-based compounds may be prudent for individual users. In all cases, consultation with local hospital guidelines regarding the frequency, nature, and acceptability of disinfection and sterilization of reusable equipment should be followed.

For devices requiring sterilization, manufacturer specifications for printing materials should be consulted. For example, 3D printed nasal swabs needed to expand testing in the US must not only be safe and provide adequate sample, but also must be sterilized and packaged appropriately for testing and eventual clinical use. Where available, limited physical impact methods such as hydrogen peroxide gas plasma or ionizing radiation may be the preferred means of sterilization, since alternative methods such as autoclaving may deform the printed parts.

## Recommendations and conclusions

We recommend that 3D printing experts communicate with their local hospital supply chain and potentially with national strategic stockpile holders. A centralized strategic local response to this crisis requires open forms of organized communication. In the United States and Canada, local and state/province supply chain experts should relay best information of what is in stock, in transit, or on backorder.

Medical devices are highly regulated for safety. While dedicated people are responding in unprecedented ways, the 3D printing community must work in parallel to ensure that emergency parts are safe, or at a minimum safer than the alternative of not using them during a pandemic. Even with the urgency of the growing COVID-19 crisis, standard safety and quality measures of 3D printing labs should continue to be followed. For larger academic medical centers that have partnerships between university-based 3D printing resources and hospitals, this is often already in place; however, appropriate safety protocols should always be reviewed.

Safe implementation of unregulated parts is essential, and risk/benefit ratios can change very rapidly as medical supplies become unavailable. Companies and regulatory bodies are strongly urged to work with the 3D printing community rapidly and efficiently. For hospital systems using internal 3D printing provided by medical or research/biomedical engineering personnel only, there is a concern for liability with 3D printing materials without safety and quality measures in place and these systems should address this concern immediately if not done already.

Intellectual property remains a concern, particularly for potentially reverse-engineering medical parts that cannot be purchased in a timely fashion during a pandemic. Given the gravity of the situation at the time of writing, it is hoped that regulators, legal experts, and policy makers can rapidly come to agreements or allowances to save human lives using the goodwill of established and needed academic-industry partnerships.

The concept of 3D printing in medicine started with the goal of improving patient education, diagnosis, and treatment [19]. We hope that this pandemic will inspire global creativity, learning and innovation through collaborative interactions of health professionals and engineers. We hope that 3D printing will be a force for a positive impact on morbidity and mortality in these trying times. Going forward, the 3D printable medical model resources described here will likely be expanded in numerous centralized model repositories with new creative open source models, descriptions of intended use, assembly instructions, and target material/printer descriptions. We hope that the readers of 3D Printing in Medicine will find this discussion useful in addressing the COVID-19 challenge and making a positive impact in patients’ lives using this transformative technology.

## References

[1] Coronavirus disease (COVID-19) Pandemic. https://www.who.int/emergencies/diseases/novel-coronavirus-2019. Accessed 24 March 2020.

[2] Ramanathan K, Antognini D, Combes A, Paden M, Zakhary B, Ogino M, MacLaren G, Brodie D, Shekar K (2020). Planning and provision of ECMO services for severe ARDS during the COVID-19 pandemic and other outbreaks of emerging infectious diseases. Lancet Respir Med.

[3] Bateman NT, Leach RM (1998). Acute oxygen therapy. BMJ.

[4] Italian hospital saves Covid-19 patients lives by 3D printing valves for reanimation devices. https://www.3dprintingmedia.network/covid-19-3d-printed-valve-for-reanimation-device/. Accessed 24 March 2020.

[5] Easy COVID-19. https://www.isinnova.it/easy-covid19-eng/. Accessed 24 March 2020..

[6] Respirator-free reanimation Venturi's valve (rev. 4). https://grabcad.com/library/respirator-free-reanimation-venturi-s-valve-1. Accessed 24 March 2020.

[7] The no2covid-ONE. https://no2covid.com. Accessed 2 Apr 2020.

[8] Code Life Ventilator Challenge. https://www.agorize.com/en/challenges/code-life-challenge?lang=en. Accessed 24 Mar 2020.

[9] WASP shares open source processes for production of personalized PPE masks and helmets. https://www.3dprintingmedia.network/personalized-ppe-mask/. .

[10] About NanoHack. https://copper3d.com/hackthepandemic/. Accessed 24 Mar 2020.

[11] HEPA Mask. https://www.thingiverse.com/thing:4222563. .

[12] Makers Guide. https://creality.com/info/makers-guide-3d-printed-face-mask-no-worries-on-mask-shortage-and-virus-infection-i00248i1.html. Accessed 24 March 2020.

[13] COVID-19 Response. https://lowellmakes.com/covid-19-response/. Accessed 24 March 2020.

[14] Flexible Mask Valvy. https://www.thingiverse.com/thing:4177128. Accessed 24 March 2020.

[15] Prusa Protective Face Shield - RC2. https://www.prusaprinters.org/prints/25857-protective-face-shield-rc1. Accessed 24 March 2020.

[16] Hands-Free Door Openers. https://www.materialise.com/en/hands-free-door-opener/technical-information. Accessed 24 Mar 2020.

[17] Guideline for Disinfection and Sterilization in Healthcare Facilities, 2008. https://www.cdc.gov/infectioncontrol/pdf/guidelines/disinfection-guidelines-H.pdf. .

[18] Technical Data Bulletin. Cleaning Reusable Respirators and Powered Air Purifying Respirator Assemblies. https://multimedia.3m.com/mws/media/988556O/tdb-cleaning-reusable-respirators-and-papr-assemblies.pdf. Accessed 24 March 2020.

[19] D'Urso PS, Atkinson RL, Lanigan MW, Earwaker WJ, Bruce IJ, Holmes A, Barker TM, Effeney DJ, Thompson RG (1998). Stereolithographic (SL) biomodelling in craniofacial surgery. Br J Plast Surg.

